# Living between stereotype and erasure: A study of the model minority myth in medical education

**DOI:** 10.1111/medu.70278

**Published:** 2026-07-19

**Authors:** Keegan D'Mello, George Kim, Robin Mackin, Amrit Kirpalani

**Affiliations:** ^1^ Lawson Health Research Institute London Ontario Canada; ^2^ Schulich School of Medicine and Dentistry Western University London Ontario Canada

## Abstract

**Background:**

Asian sub‐ethnic trainees and physician educators constitute a substantial proportion of the medical workforce across Western countries yet remain systematically excluded from diversity and inclusion discourse. The model minority myth (MMM)—the construct that positions Asians as uniformly high‐achieving, non‐confrontational and culturally assimilated—operates as a structural force that simultaneously confers partial privilege and constrains identity, advancement and help‐seeking. Despite its harms, the mechanisms by which the MMM operates across medical training remain understudied.

**Methods:**

Fourteen semi‐structured interviews were conducted with Asian sub‐ethnic medical students, residents and physician educators across medical training institutions. A hermeneutic phenomenological approach was used, guided by an Asian Critical Theory (AsianCrit) framework. Interviews were audio‐recorded, transcribed and analysed using deductive and inductive thematic analysis to ensure rigour.

**Results:**

Six themes were identified: (1) internalised exceptionalism and family‐mediated career pressure; (2) everyday racial misrecognition and erasure, including name mispronunciation and physical interchangeability; (3) structural barriers to leadership, including the bamboo ceiling, voluntold labour and social exclusion; (4) homogenisation and the use of the MMM as a wedge against Black and Indigenous peers; (5) performative equity, diversity and inclusion (EDI) initiatives, curriculum gaps and tokenistic institutional participation; and (6) mentorship patterns shaped by shared ethnic and cultural experience. Cross‐cutting findings included the MMM's suppression of help‐seeking, the differential operation of the myth across Arab/Middle Eastern sub‐ethnic identities and the influence of geopolitical events on identity expression.

**Conclusions:**

The MMM shapes lived experience across the full arc of medical training, from admissions through senior leadership. Its harms are not uniform: They vary by sub‐ethnic group, generation, gender and institutional context. Medical education must move beyond performative EDI to structurally address the MMM's manifestations in curriculum, selection processes and mentorship. Disaggregating ‘Asian’ into its constituent sub‐ethnic identities is essential to equitable policy and practice.

## Abstract 2

Asian medical trainees carry the weight of the model minority myth from day one ‐ shaping career pressure, stifling help‐seeking, and closing doors to leadership. #MedEd #HealthEquity #AsianCrit

## 1 INTRODUCTION

The model minority myth (MMM)—the construct that positions Asian communities as uniformly educated, hardworking and socioculturally assimilated and therefore immune to racial harm—occupies a peculiar place in medical education's equity landscape. Asian‐identifying individuals constitute approximately one in five medical students and physicians in the United States,^1^ and representing the largest visible minority group in Canadian medical schools,^2^ and are the largest visible minority group in Canadian medical schools,^2^, ^3^ This paradox is animated, in large part, by the MMM—the racialised social construct that frames Asian communities as uniformly educated, hardworking and socioculturally assimilated and therefore immune to the harms that accompany minority status.^4^, ^5^

The MMM was not an organic cultural observation but a politically constructed narrative. It emerged in the United States during the Civil Rights Movement as a rhetorical device to undercut claims of systemic racism by positioning Asian immigrants—selectively drawn from professional and educated classes following immigration policy changes in the 1960s—as evidence that racism could be overcome through individual effort.^6^ Parallel processes operated in Canada, the United Kingdom and Australia, where immigration selection criteria that favoured skilled professionals, combined with histories of explicit exclusion and then managed admission, produced a demographically skewed pool that reinforced the myth of inherent Asian medical aptitude.^2^

Empirically, the MMM is demonstrably false on multiple dimensions. Sub‐ethnic disaggregation reveals profound disparities in representation: Southeast Asian communities, including those of Vietnamese, Cambodian and Laotian descent, remain severely underrepresented in medicine relative to their population share, masked by aggregate ‘Asian’ data.^7^, ^8^, ^9^ Despite this evidence, Asian trainees and physicians are rarely the primary focus of EDI initiatives, and the specific mechanisms by which the MMM operates across medical training environments remain understudied qualitatively.

Asian Critical Theory (AsianCrit), developed by Museus and Iftikar,^7^ provides the theoretical framework for this inquiry. AsianCrit was built on the recognition that existing Critical Race Theory frameworks did not adequately account for how Asian communities are racialised in distinct and contradictory ways: simultaneously elevated and constrained. All seven of its tenets informed the deductive coding framework; three are foregrounded here because they generated the most analytically prominent findings. Asianisation describes how Asian communities are racialised through stereotypes, including the MMM, that position them as model, compliant and culturally assimilable; the MMM is thus one specific mechanism of Asianisation rather than a separate concept. Transnational contexts attend to how conditions in countries of origin and diasporic political events shape the racialisation of Asian‐origin communities in their countries of settlement. Commitment to social justice shaped our analytic orientation: We attended not only to accounts of harm but also to the strategies through which participants navigated and, at times, disrupted the conditions the MMM produces.

Although quantitative analyses have documented workforce disparities and microaggression prevalence,^1^, ^4^, ^8^, ^9^ and although discourse analyses have examined the MMM in pre‐medical communities,^3^ no qualitative study has examined how the MMM is experienced across the full continuum from medical student through senior physician educator nor attended to the heterogeneity of Asian sub‐ethnic experience within that continuum. This study aimed to characterise how Asian sub‐ethnic trainees and physician educators experience the MMM across the full arc of medical training, identify the structural contexts in which it manifests, document both the harms it produces and the strategies through which participants navigated and disrupted it and generate findings that can inform equitable educational policy and practice.

## 2 METHODS

### 2.1 Study design

Hermeneutic phenomenology provided the interpretive paradigm for this study: It attends to how individuals make meaning of experience while acknowledging that the researcher's perspective shapes interpretation rather than distorting it.^10^ This orientation is well suited to inquiry into racialised experience, which is often tacit, cumulative and only partially articulable by participants. AsianCrit served as the theoretical framework, shaping both what we looked for and how we interpreted it. Thematic analysis provided the procedural structure for systematic coding and theme development.^7^

### 2.2 Participants

Purposive and snowball sampling were used to recruit participants based in Canada, members of the Asian diaspora training or working within the Canadian medical system, across three roles: medical students (clerkship), residents (any postgraduate year) and physician educators (any level of seniority). Eligible participants identified as Asian sub‐ethnic—a deliberately inclusive criterion that encompassed South Asian, East Asian, Southeast Asian and Arab/Middle Eastern participants, reflecting AsianCrit's commitment to strategic (anti)essentialism and the recognition that ‘Asian’ encompasses diverse and incommensurable identities. Within these groupings, specific countries of origin or heritage were recorded during interviews; these are not reported at the individual level to protect confidentiality but informed the sub‐ethnic analysis throughout. Participants were recruited through professional networks, medical school associations and peer referral. Fourteen participants were enrolled; characteristics are summarised in Table 1.

**TABLE 1:** Participant characteristics (*N* = 14).

ID	Role	Sub‐ethnic group	Training stage	Primary themes raised
P01	Physician educator	East Asian	Senior faculty	MMM internalisation; EDI tokenism; career pressure
P02	Senior leader	East Asian	Senior faculty	Bamboo ceiling; gender–race intersectionality; survivor bias
P03	Resident	South Asian	PGY5–6	Name mispronunciation; geographic variation in identity expression
P04	Physician educator	East Asian	Mid‐career faculty	Racial misrecognition; perpetual foreigner; social exclusion
P05	Physician educator	Southeast Asian	Mid‐career faculty	Conflict avoidance stereotype; compliant Asian archetype
P06	Physician educator	South Asian	Early career faculty	Career pressure; performative EDI; South Asian faculty dynamics
P07	Physician educator	Southeast Asian	Senior faculty	Compliant femininity; informal support networks; EDI fatigue
P08	Physician educator	South Asian	Mid‐career faculty	Exclusion from ‘diversity’; work twice as hard ethos
P09	Resident	South Asian	PGY1	Differential preceptor dynamics; family career pressure
P10	Medical student	East Asian	Clerkship	Name erasure; Confucian hierarchy; performative EDI
P11	Physician educator	South Asian	Mid‐career faculty	MMM benefit; tokenistic committee recruitment; CSA stigma
P12	Resident	South Asian	PGY1	Stereotype deflection; identity aversion; EDI inauthenticity
P13	Resident	Arab/Middle Eastern	PGY2	Identity suppression; geopolitical fear; subjectivity in selection
P14	Resident	South Asian	PGY1	Racial ambiguity; phenotypic hierarchy; EDI classroom failure

*Note*: Sub‐ethnic groups are aggregated (South Asian; East Asian; Southeast Asian; and Arab/Middle Eastern) to protect participant confidentiality. Role and training stage descriptors have been generalised for the same reason.

Abbreviations: CSA, Canadian student who studied abroad; PGY, postgraduate year.

### 2.3 Data collection

Semi‐structured interviews were conducted via videoconference by a trained research team member of Asian sub‐ethnic background using an interview guide developed by the research team. The interview guide introduced the MMM by name and provided a brief contextual definition at the outset of each interview; participants were not required to be familiar with the term prior to participation, and several encountered it formally for the first time during the session. The guide addressed the influence of racial identity on medical training and career development; experiences of stereotyping, microaggression and racial misrecognition; perceptions of EDI initiatives and curriculum; mentorship patterns; and the role of geopolitical and cultural events. Interviews lasted 35–65 min were audio‐recorded and transcribed verbatim. All transcripts were reviewed for accuracy and de‐identified prior to analysis.

### 2.4 Analysis

Thematic analysis was conducted using a framework approach informed by AsianCrit tenets.^7^, ^10^ Initial coding was deductive, drawing on the seven AsianCrit tenets (Asianisation, transnational contexts, reconstructive history, strategic anti‐essentialism, intersectionality, story/theory/praxis and commitment to social justice) and inductive, allowing themes to develop inductively from the data. Two team members independently coded four transcripts, and consensus was reached through discussion. Final themes were developed through iterative review across the full team. Reflexive memos were maintained throughout to document interpretive decisions and positionality. The thematic write‐up foregrounds the tenets most analytically prominent in the data; all seven informed the coding process but not all generated findings of sufficient weight to warrant theme‐level discussion.

### 2.5 Reflexivity

The research team comprised one researcher, who conducted all interviews, and three physician educators, including the principal investigator. The team holds South Asian and East Asian sub‐ethnic identities, reflecting AsianCrit's commitment to centring Asian voices while attending to the institutional contexts in which those voices operate. The researcher's role as a non‐faculty research professional of Asian sub‐ethnic background facilitated candour; structured reflexive journals after each interview guarded against assumed understanding replacing genuine inquiry and were reviewed at team check‐ins to inform iterative revisions to the interview guide.

### 2.6 Ethical considerations

Ethics approval was obtained from the authors' university Research Ethics Board. All participants provided written informed consent. Participant confidentiality was protected through removal of all identifying details, pseudonymisation and aggregation of sub‐ethnic identities in reporting.

## 3 RESULTS

Fourteen participants contributed data across a range of training stages, sub‐ethnic backgrounds and institutional contexts (Table 1). Six major themes were identified through analysis; representative quotations are summarised in Table 2. Where participants explicitly named the MMM, this is noted; where the connection is interpretive, grounded in AsianCrit analysis, this is signalled. Themes 1 through 5 address the structural contexts in which the MMM manifests; Theme 6 and cross‐cutting findings address how participants navigated and disrupted it. Table 1 notes the career stage of each participant, allowing readers to trace career stage to specific quotes and themes.

**TABLE 2:** Major themes, sub‐themes and representative quotations.

Theme	Sub‐themes	Representative quotation
1. Internalised exceptionalism and family‐mediated pressure	Medicine/engineering binary; intra‐community surveillance (‘why can't you be more like X’); generational gradient	‘The idea that you have to be extremely hardworking and ambitious … I do not think I would have recognized it as a stereotype being put on me’. (P11)
2. Racial misrecognition and erasure	Name mispronunciation; physical interchangeability; perpetual foreigner construction	‘It transitioned from these series of microaggressions that every Asian person felt to actual aggression’. (P01, paraphrase)
3. Bamboo ceiling and structural barriers	Work twice as hard ethos; voluntold labour; compliant femininity; social exclusion	‘It's easier to ascend if you're compliant and non‐disruptive. But then you've trapped yourself’. (P07)
4. Homogenisation and inter‐minority hierarchy	Pan‐Asian erasure; Arab/Muslim political marginalisation; MMM as wedge against Black/Indigenous peers	‘I've heard: “that person totally wouldn't have met the cutoffs for the regular stream.”’ (P13)
5. EDI, curriculum, and tokenism	Absent Asian history; performative EDI; professional competencies as site of harm; tokenistic recruitment	‘Our EDI sessions are not necessarily contributing to our wellness or betterment of EDI practices’. (P13)
6. Mentorship and the pull of shared experience	Same‐background mentorship; limits of ethnic similarity; geographic variation in access	‘The perfect mentor would be a South Asian woman, in community medicine, who can talk about racism and work‐life balance’. (P14)

*Note*: Quotations are verbatim excerpts from interview transcripts. Participant identifiers (P01–P14) correspond to Table 1. Minor identifying details within quotations have been removed or generalised.

Abbreviations: EDI, equity, diversity and inclusion; MMM, model minority myth.

### 3.1 Theme 1: Internalised exceptionalism and family‐mediated career pressure

Almost universally, participants described environments in which medicine was the unquestioned horizon of acceptable ambition. This expectation was transmitted most directly through immigrant parents whose own trajectories involved substantial professional sacrifice and downward mobility upon arriving in Canada.

It's hard not to think of it as coming from seeing both my parents coming in from India in the seventies and having to work hard … I recognized that there's probably a level of privilege I've been given by the hard work my parents did. (P11)

Some participants named the MMM directly when describing family career pressure; others described the same dynamic without connecting it explicitly to the myth. The MMM also functioned as intra‐community surveillance: Participants described being measured against high‐achieving Asian peers and feeling pressure to emulate their success, a recursive structure in which MMM‐compliant individuals become instruments of pressure on others. Importantly, several described their compliance not as chosen but as absorbed over years of a medical training environment that rewarded it.

I definitely benefited from the model minority myth … but I know it's hindered other people because it's created unnecessary pressure on them that affected their ability to choose a profession they actually want. (P11)

### 3.2 Theme 2: Racial misrecognition and everyday erasure

Participants described patterned, iterative experiences of being misidentified, physically interchanged with other Asian colleagues and having their names mispronounced or anglicised without consent. Name erasure emerged as a symbolically charged site: Participants described a learned resignation, an accommodation they later named explicitly as a manifestation of the MMM's demand for non‐confrontation.

There's this feeling of—I always joke it's hard to know the difference between being an immigrant and being Canadian, because you always want to have that level of ‘let me not rock too much’. But this is the model minority myth right there. (P11)

Being confused with the only other Asian colleague of similar gender, irrespective of physical resemblance, was described by multiple participants over extended career trajectories. Patient assumptions that Asian physicians were nurses were similarly common. One participant described differential patient trust based on perceived race: When read as Indian, the MMM's competence archetype activated and trust increased; when the same participant was read as Black, it did not. This account illustrates that the MMM does not operate only on Asian individuals but functions as a racial hierarchy, conferring and withholding trust across racial lines. Being confused with the only other Asian colleague of similar gender, irrespective of physical resemblance, is itself a product of Asianisation, the same process that flattens diverse Asian sub‐ethnic identities into a single stereotype. A counterstory ran alongside these accounts: several participants described becoming deliberate about asserting their credentials and cultural knowledge in patient encounters, not as resistance to the MMM per se, but as a form of reclamation.

### 3.3 Theme 3: The bamboo ceiling and structural barriers to advancement

Where Theme 2 documented interpersonal misrecognition, Theme 3 addresses its structural consequences, showing how the MMM operates at both relational and institutional levels. The gap between high performance and leadership attainment was identified as a structural reality by faculty participants and as an anticipated trajectory by trainees. Participants used variations of ‘work twice as hard to be half as good’—a phrase often traced explicitly to parents or community elders—to articulate the asymmetry between effort and recognition. Several described being ‘voluntold’: assigned to committees without recognition or compensation. Participants did not uniformly name the bamboo ceiling explicitly; rather, the authors interpreted these accounts through AsianCrit's tenet of Asianisation, the process by which structural expectations become attributed to cultural character. The connection to AsianCrit's Asianisation tenet is direct: Because the MMM constructs Asian identity as naturally compliant, institutions can penalise assertive Asian faculty without recognising this as discrimination. Structural exclusion gets attributed to cultural fit rather than to bias, rendering the ceiling invisible to those who impose it.

It's easier to ascend if you're compliant and non‐disruptive. But then you've trapped yourself. (P07)

Female participants described a compounded dynamic: conforming to the compliant archetype enabled superficial advancement while foreclosing authentic leadership, and several described informal racialised women's networks as a grassroots response.

Social exclusion through culturally specific activities, sports leagues, golf events and sports‐dominated conversational norms was identified as a mechanism by which informal professional networks were constituted in ways that positioned Asian colleagues as peripheral.

### 3.4 Theme 4: Homogenisation, sub‐ethnic diversity and the MMM as inter‐minority wedge

The pan‐ethnic ‘Asian’ category was experienced as an institutional and social imposition that erased meaningful sub‐ethnic difference. Participants described being assigned intelligence, technical competence or docility on the basis of perceived Asian identity, irrespective of their specific heritage. Students who trained abroad faced dual MMM framings: either deficient merit or inherited privilege, both denying individual circumstance.

One participant who identified as Arab/Middle Eastern described an experience that diverged structurally from the South and East Asian MMM narratives. Following the escalation of the Gaza conflict in late 2023, this participant described suppressing their Arab and Muslim identity with supervisors and near‐peers, avoiding advocacy related to the conflict for fear of professional retaliation and observing colleagues at other institutions where identity suppression was even more thorough. This account illustrated that the MMM does not affect all Asian sub‐ethnic groups symmetrically: Political events can shift particular sub‐ethnic identities from ‘model’ to ‘suspect’, and EDI commitments may fail to protect trainees precisely when protection is most needed. This account is interpreted through AsianCrit's tenet of transnational contexts: The political conditions of Asian‐origin communities abroad can rapidly reconfigure their racialisation within institutions of settlement.

In the last year, I've felt a shift—both for myself and my colleagues from the Middle East—in how forthcoming we are about our identity, because of fear of retaliation. (P13)

Comments positioning Black and Indigenous applicants as less deserving of dedicated admissions pathways, on the grounds that their admission standards were lower, were described by multiple participants, usually attributed to colleagues rather than faculty. The logic reproduced the MMM's wedge function precisely: Asian hard work and merit were implicitly contrasted with pathways framed as special treatment for others, reinforcing racial hierarchy rather than solidarity.

### 3.5 Theme 5: Performative EDI, curriculum gaps and tokenism

A convergent finding across trainee and educator participants was frustration with EDI initiatives perceived as performative, designed to signal institutional commitment rather than produce structural change. This pattern reflects AsianCrit's commitment to social justice: the gap between institutional rhetoric and structural action. The near‐total absence of Asian historical experience from formal curricula was noted: Immigration restriction histories, anti‐Asian exclusion legislation, South Asian exclusion policies and Southeast Asian refugee histories were absent from participants' medical education, reinforcing the perpetual foreigner construction, the racialised assumption that Asian individuals are inherently foreign regardless of citizenship, language or generational status.

Professional competencies sessions addressing racism were described by trainee participants as sites of potential harm when facilitated by older white faculty in predominantly white small groups, producing declarations of non‐racism rather than substantive engagement. One participant described sitting in silence as peers went around a circle identifying which racism indicators did not apply to them, whereas the participant—the only person of colour in the room—did not feel safe disclosing that most of them did.

To be in that room where that is my mentor—the person supposed to be teaching me how to unpack racism—and he's saying discrimination doesn't happen anymore. That's the person I'm supposed to be looking up to. (P14)

Educator participants described tokenistic committee recruitment without genuine participation and noted that unrecognised EDI labour drove racialised faculty to withdraw from this work entirely. Yet withdrawal was not the only response: Some described finding lateral communities, informal peer groups of racialised faculty, where the work they were denied in formal structures continued on their own terms. This reflects the liminal position that runs through each theme: Asian medical faculty and trainees are simultaneously expected to represent diversity and denied the institutional conditions to do so authentically.

### 3.6 Theme 6: Mentorship and the gravitational pull of shared experience

A majority of participants described gravitating towards mentors who shared aspects of their racial, ethnic or cultural background, not as an exclusive preference, but as a meaningful facilitating factor. This reflects AsianCrit's tenet of reconstructive history, participants' own histories of navigating exclusion as a basis for navigating current systems. Seeing someone who ‘looked like them’ in a senior role was experienced as evidential: proof that a particular career trajectory was navigable. For those in educator roles, the reverse pattern was also noted, with mentees of similar backgrounds actively seeking them out.

The perfect mentor would be a South Asian woman who works in community medicine, can balance family and leadership, has faced racism and can talk about it—someone doing exactly what I want to do. (P14)

Critiques of same‐background mentorship were also noted: Second‐generation participants observed that ethnic similarity did not guarantee experiential resonance. Geographic variation was pronounced: Participants in diverse urban centres had substantially greater access.

## 4 DISCUSSION

This study provides the first empirical qualitative account of the MMM's operation across the full continuum of medical training, from student through senior physician educator, attending to the heterogeneity of Asian sub‐ethnic experience. Our findings extend the existing literature in several important ways and converge with it in others. These mechanisms share features with racialised harm documented for other minoritised groups, including stereotype threat and identity taxation, but the MMM's distinguishing feature is its dual structure: It simultaneously elevates and constrains, making resistance costly in ways that more overtly negative stereotypes do not.

This study provides the first empirical qualitative account of the MMM's operation across the full continuum of medical training, from student through senior physician educator, attending to the heterogeneity of Asian sub‐ethnic experience. Our findings extend the existing literature in several important ways and converge with it in others. Across career stages, name erasure, racial misrecognition and internalised exceptionalism were broadly shared. Structural themes diverged: trainees described anticipated barriers and identity suppression under evaluation, whereas faculty accounts centred on realised exclusion from leadership and the cumulative toll of EDI work without recognition. Mentorship was the only theme where career stage produced a reversal; trainees sought same‐background mentors, whereas educators described being sought out, illuminating a relational dynamic that operates differently depending on position in the hierarchy. These patterns confirm that the MMM operates differently depending on where one sits in the institutional hierarchy and illustrate the added value of studying it across the full trainee‐to‐educator continuum.

The bamboo ceiling has been documented quantitatively in US academic medicine, with Asian physicians consistently underrepresented in leadership relative to their faculty representation.^8^, ^11^ Our data illuminate the mechanisms: penalisation of assertiveness, informal social exclusion and compulsory non‐confrontation. These are structural effects of an institutionalised racial stereotype. Targeted strategies for mentorship and sponsorship, distinct from tokenistic committee recruitment, have been proposed as structural interventions.^12^

The microaggression literature has documented the prevalence and impact of racial incidents experienced by Asian American medical students: approximately 70% of surveyed students report at least one incident during training, with perpetration distributed across patients, peers and faculty.^9^ Our findings are consistent with this picture and extend it by attending to the accumulated, cumulative character of these experiences across career trajectories, a dimension that cross‐sectional surveys cannot capture. The resignation with which many participants described name mispronunciation is notable. Several connected this directly to the MMM's demand for non‐confrontation; for others, the normalisation was not named as such, but the pattern was consistent across accounts. These dynamics connect to broader scholarship on stereotype threat and racial trauma: Vigilance and performance anxiety among racialised learners are intensified by the MMM's simultaneous demand for exceptionalism and invisibility. These dynamics connect to broader scholarship on stereotype threat and racial trauma: Vigilance and performance anxiety among racialised learners are intensified by the MMM's simultaneous demand for exceptionalism and invisibility.^13^, ^14^

The Arab/Middle Eastern participant's experience extends this literature: AsianCrit's transnational contexts tenet is essential for interpreting how Arab and Muslim identity was reconfigured from ‘model’ to suspect, illustrating that the MMM responds to political conditions.^7^, ^15^ Post‐October 7 identity suppression in clinical training represents a form of racial harm that existing MMM frameworks do not adequately capture.

The performative EDI finding converges with growing critiques of diversity initiatives in medical education that prioritise symbolic gestures over structural change.^16^ The professional competencies classroom as a site of harm, rather than learning, has particular implications for curriculum design: The presence of content addressing racism does not guarantee outcomes that support racialised trainees. Facilitator composition, group demographics, psychological safety and the power asymmetry between faculty and students all require explicit attention. Our data suggest that the current approach, in many institutions, may actively reinforce the MMM by providing a space for majority‐group members to declare themselves non‐racist without substantive engagement with how racial stereotypes operate structurally. Taken together, Themes 5 and 6 illuminate AsianCrit's commitment to social justice: the gap between performative inclusion and structural change and the grassroots relational infrastructure that racialised faculty and trainees build in its absence.

Several participants described avoiding disclosure of academic difficulty or personal distress, attributing this directly to the internalised expectation that struggle was incompatible with their identity as an Asian high‐performer. The MMM's suppression of help‐seeking represents an urgency that has received insufficient attention.^17^ Asynchronous, anonymous and culturally attuned support pathways are not a luxury but a patient safety issue.

### 4.1 Implications for practice

The six themes generate distinct practice implications. AsianCrit was developed to describe the racialisation of Asian communities in North America, and the applicability of these findings to other contexts requires local interpretation; we offer these not as universal prescriptions but as directions that the data support. Curriculum should incorporate Asian historical experience, including exclusion histories, as substantive content across Western medical education systems. EDI programming requires rigorous attention to facilitation training and group composition. Admissions and residency selection should be examined for MMM‐shaped evaluator bias, with sub‐ethnic outcome monitoring. Mentorship programming should facilitate same‐background connections while protecting racialised faculty from disproportionate EDI labour. Institutions should develop disaggregated data systems distinguishing Asian sub‐ethnic groups. Institutions should also consider whether accelerated attention to Asian sub‐ethnic equity risks zero‐sum framing; implementation should be accompanied by messaging that equity for Asian trainees is not in competition with initiatives for other racialised groups. Whether these patterns and responses apply in non‐North American medical education systems is an important question for future comparative work.

### 4.2 Strengths and limitations

The primary strength of this study is its AsianCrit framework, enabling connections between individual experience and structural dynamics that purely inductive approaches might miss. The sample's inclusion of Arab/Middle Eastern participants enriches and complicates the dominant East/South Asian framing of the MMM. The study's hermeneutic approach, with explicit positionality reflexivity and co‐coding, supports rigour within a qualitative paradigm.

Limitations include the institutional concentration of participants within a small number of proximate medical schools, which may reduce transferability. Intersectional demographic data, including gender identity, religion, generational status and socioeconomic background, were not systematically collected, limiting interpretation of how overlapping social positions shaped MMM experiences. The study was conducted partly during the period of heightened anti‐Arab and anti‐Muslim sentiment following the October 2023 escalation of conflict in Gaza, which may have heightened the salience of Arab/Middle Eastern identity for some participants and shaped their accounts in ways that do not reflect their typical day‐to‐day experience of medical training. As a study of self‐reported experience, it captures perceptions and interpretations of structural dynamics rather than providing direct evidence of those dynamics. Future work should examine the MMM's operation using observational, longitudinal or administrative data to triangulate the experiential accounts reported here.

### 4.3 Conclusions

The MMM is not a relic of historical racism nor a harmless compliment. It is a structural force that operates across medical education—shaping who is silenced, who is credited, who is promoted and who seeks help. Its harms are distributed unequally by sub‐ethnic group, generation, gender and institutional context. The patterns documented here reflect structural conditions common to medical training across Western contexts, not a single national system. Addressing the MMM requires structural reform of the spaces through which racial hierarchies in medicine are reproduced. This study provides an empirical foundation for that work.

## AUTHOR CONTRIBUTIONS

**Keegan D'Mello:** Project administration; data curation; formal analysis; methodology; writing—original draft; writing—review and editing; investigation. **George Kim:** Formal analysis; methodology; writing—review and editing; investigation. **Robin Mackin:** Methodology; formal analysis; writing—review and editing; investigation. **Amrit Kirpalani:** Methodology; formal analysis; writing—review and editing; writing—original draft; conceptualization; investigation; funding acquisition; supervision.

## CONFLICT OF INTEREST STATEMENT

The authors have no competing interests or conflicts to disclose.

## ETHICS STATEMENT

Ethics approval was obtained from the Western University Health Sciences Research Ethics Board (Project ID 125306). All participants provided written informed consent. Participant confidentiality was protected through removal of all identifying details, pseudonymisation, and aggregation of sub‐ethnic identities in reporting.

## Data Availability

The data that support the findings of this study are available on request from the corresponding author. The data are not publicly available due to privacy or ethical restrictions.
